# Fatigue in osteoarthritis: a qualitative study

**DOI:** 10.1186/1471-2474-9-63

**Published:** 2008-05-01

**Authors:** J Denise Power, Elizabeth M Badley, Melissa R French, Angela J Wall, Gillian A Hawker

**Affiliations:** 1 Department of Public Health Sciences, University of Toronto, Toronto, Ontario, Canada; 2 Arthritis Community Research and Evaluation Unit, Toronto Western Research Institute Toronto, Ontario, Canada; 3 Canadian Osteoarthritis Research Program, Women's College Hospital, Toronto, Ontario, Canada; 4 Department of Health Policy, Management and Evaluation, University of Toronto, Toronto, Ontario, Canada; 5 Department of Medicine, Division of Rheumatology, University of Toronto, Toronto, Ontario, Canada

## Abstract

**Background:**

Fatigue is recognized as a disabling symptom in many chronic conditions including rheumatic disorders such as rheumatoid arthritis (RA) and lupus. Fatigue in osteoarthritis (OA) is not routinely evaluated and has only been considered in a very limited number of studies. To date, these studies have focused primarily on patients with OA under rheumatological care, which represent the minority of people living with OA. The purpose of this study was to increase our understanding of the fatigue experience in community dwelling people with OA.

**Methods:**

In 2004, 8 focus groups were conducted with 28 men and 18 women (mean age 72.3) with symptomatic hip or knee OA recruited from a population-based cohort. Participants completed a self-administered questionnaire, which included demographics, measures of OA severity (WOMAC), depression (CES-D) and fatigue (FACIT). Sessions were audio taped and transcribed verbatim. Two researchers independently reviewed the transcripts to identify themes. Findings were compared and consensus reached.

**Results:**

Mean pain, disability, depression and fatigue scores were 8.7/20, 27.8/68, 15.4/60, and 30.9/52, respectively. Participants described their fatigue as exhaustion, being tired and "coming up against a brick wall". Participants generally perceived fatigue as different from sleepiness and distinguished physical from mental fatigue. Factors believed to increase fatigue included OA pain and pain medications, aging, various types of weather and poor sleep. Mental health was identified as both affecting fatigue and being affected by fatigue. Participants described fatigue as impacting physical function, and their ability to participate in social activities and to do household chores. Rest, exercise, and avoiding or getting assistance with activities were cited as ways of coping. Participants generally did not discuss their fatigue with anyone except their spouses.

**Conclusion:**

Participants with OA described experiencing notable amounts of fatigue and indicated that it had a substantial impact on their lives. Further research is required to better understand the role of fatigue in OA in order to identify strategies to reduce its impact.

## Background

Arthritis is one of the most common chronic conditions in the population[1], resulting in substantial social and economic costs [2]. Due to the aging of the population, the prevalence and impact of this disease is projected to greatly increase [3,4]. Osteoarthritis (OA) is the most common form of arthritis, accounting for approximately 75% of the disease [5] and ranking among the top ten causes of disability worldwide [6]. In 2003, Canadians living with arthritis identified fatigue as a research priority [7], but to date, very little is known about the role of this symptom in OA.

Fatigue is a common, non-specific, symptom experienced by most people at some point during their lives. It is the enduring, subjective sensation of generalized tiredness or exhaustion [8]. It is also conceptualized variously as weariness, weakness and depleted energy [9]. Fatigue is distinguished from sleepiness [9]and can be divided into that which is a primary complaint or a secondary complaint due to a known disease [10].

Many chronic physical illnesses are associated with fatigue, including cancer [11], and multiple sclerosis [12]. Fatigue has also been recognized as an important symptom in rheumatic disorders such as systemic lupus erythematosus and rheumatoid arthritis (RA) [13,14]. Fatigue is often identified as one of the most challenging aspects of chronic disease [8,15]. It is a disabling symptom that has been shown to have a substantial impact on patients' self-care activities [16] and overall quality of life [17,18].

Fatigue in OA is not routinely evaluated and has only been considered in a limited number of studies. To date, these studies have focused primarily on patients with OA under rheumatological care, which represents the minority of people living with OA. However, among this subgroup with OA, fatigue levels have been reported as similar to those of patients with RA [19-24]. Wolfe et al [20] reported that approximately 40% of OA patients have substantial fatigue and, as for RA, fatigue in OA is associated with pain, sleep disturbance and depressed mood [20,23]. Gignac et al [25] recently reported that adults with moderate OA participating in focus groups describe their fatigue as debilitating and occasionally activity restricting.

The purpose of our qualitative study was to increase our understanding of the fatigue experience in community-dwelling individuals with hip and knee OA. This study is the first step in a program of research examining fatigue in OA and how it relates to OA symptoms such as pain and disability, as well as mood and sleep. Better understanding fatigue and how it relates to these symptoms has the potential to lead to better management strategies and improved quality of life for people living with OA.

## Methods

Most participants were recruited from an existing population-based cohort of individuals with at least moderately severe symptomatic hip or knee OA residing in two counties of Ontario, Canada. Extensive details on cohort design have been published elsewhere [26,27]. Briefly, eligible participants in the two counties were English-speaking adults aged 55+ years who indicated on a screening survey, conducted between January 1996 and October 1998, that they had at least moderately severe hip or knee complaints defined as meeting all of the following criteria: (1) difficulty in the past 3 months with each of stair climbing, arising from a chair, standing and walking, and (2) self-reported swelling, pain, or stiffness in any joint lasting ≥ 6 weeks in the past 3 months and (3) indication on a homunculus that a hip and/or knee was "troublesome". To limit the study to those with OA, individuals were deemed ineligible if they indicated they had been diagnosed with or were prescribed medication for inflammatory arthritis.

As recruitment for the fatigue focus groups was done simultaneously with recruitment for analogous groups on pain, data on participation rates for the two types of groups could not be separated. Individuals with other conditions closely linked to fatigue or pain were not eligible. One hundred and fifty-five cohort members were asked to participate in the focus groups, as well as 5 individuals who were not part of the original cohort. These latter individuals were between the ages of 55 and 63 years and were included to assist in capturing a broader range of experiences, as the youngest members of the cohort were 64 years of age at the time of recruitment. These "new" participants were recruited from arthritis patient organizations, other research groups or had indicated a willingness to participate in research at dissemination events. These participants met the same eligibility criteria as for the cohort. Of the 160 individuals in total thus asked to participate, 87 (54.4%) attended one of the sessions, 6 (3.9%) did not show for their scheduled session, 48 (31.0%) were unavailable and 19 (12.3%) cancelled with the intention of rescheduling, but the groups were completed prior to this occurring. Participants were scheduled to attend either a fatigue or pain focus group based on their availability, as well as their sex, age and urban/rural status so as to ensure that approximate balance on these variables was achieved.

We conducted focus groups on fatigue in OA, rather than individual interviews, in order to encourage participants to discuss and compare their experiences, allowing for a comprehensive description of osteoarthritis fatigue to be captured. Focus groups were comprised of 3–8 participants and were conducted until we felt "saturation" (no new themes identified) was reached. Groups were conducted separately with men and women in order to encourage open discussion around topics that participants might be more hesitant to discuss in a mixed gender environment, such as the impact of fatigue on mood and relationships. Groups were also age-stratified (> 75, 65–75, < 65), where possible. Participants provided informed, written consent before participation. The Research Ethics Board of Sunnybrook and Women's College Health Sciences Centre approved the study (Reference Number 091-2004).

All focus groups were conducted between May and October 2004 and lead by a single moderator. Identical questions were asked of each group (Table 1) in sessions lasting 2–2.5 hours. Each focus group was audio taped, transcribed verbatim and imported into N6 software. At the start of each focus group, participants completed a questionnaire that included age, sex, education and race, as well as the following measures:

**Table 1:** Focus Group Questions

1. Tell us about the fatigue you experience with your arthritis. Do you ever feel fatigued? What is it like?
2. When you think of fatigue and sleepiness, do you see them as different? How?
3. What do you think causes your fatigue?
4. How does your arthritis affect your fatigue?
5. What, if anything, has an effect on your fatigue? What makes you more fatigued/less fatigued?
6. What, if anything, has been the impact of this fatigue on your life?
7. What changes or modifications have you made to cope with your fatigue?
8. Have you noticed any relationship between your fatigue and how you feel including changes in other symptoms or changes in emotions or mood? [If they see as related: Tell me more, does one come first, does one lead to the other or one cause the other for you?]
9. Do you talk about your fatigue with others? With who? What do you tell them?
10. We all have experiences of fatigue in our lives due to various problems. What makes your arthritis fatigue different from other kinds of fatigue?

### Western Ontario and McMaster Universities Osteoarthritis Index (WOMAC)

Data were collected to assess OA severity in participants using the 5-item pain and 17-item physical functioning subscales, which assess the amount of pain and functional limitation experienced in the past week while performing various activities [28]. The WOMAC has been shown to be valid, reliable and responsive [28-30].

### Functional Assessment of Chronic Illness Therapy Fatigue Scale (FACIT)

This 13- item instrument provides a measure of overall fatigue and was included to supplement the qualitative fatigue data collected and aid in assessing the severity of fatigue experienced by participants. The FACIT assesses the extent of self-reported tiredness, weakness and difficulty conducting usual activities due to fatigue in the previous week. The FACIT-F has been used and validated in a variety of populations, including inflammatory arthritis [31,32].

### Center for Epidemiologic Studies Depression Scale (CES-D)

This 20-item instrument measures the frequency of depressive symptoms in the previous week [33] and was included to further characterize participants in light of the strong associations between depression and fatigue. The CES-D has been validated across a variety of demographic characteristics, including in the elderly [34,35].

## Analyses

Focus group transcripts were independently reviewed by two of the study authors (JDP, MRF) to identify and code distinct themes. Themes were compared and discussed until consensus was reached. A list of themes and exemplars of each were then identified. Descriptive statistics (percentages, means, medians etc.) were generated to summarize the demographic characteristics of the cohort as well as scores on the questionnaire measures. These analyses were conducted using Statistical Package for the Social Sciences (SPSS) version 14.0.

## Results

The eight focus groups included 28 (60.9%) women and 18 (39.1%) men (Table 2). All participants were Caucasian and ranged in age from 56 to 88 years (mean ± SD = 72.3 ± 7.7 years). Exactly half (50.0%) had a highschool education with 26.2% and 23.8% having less than and greater than a high school education, respectively. Participants had moderate WOMAC pain and physical functioning scores (Table 3). CES-D scores were right-skewed, with a mean and median of 15.4 and 14.5, respectively. Seventeen participants (37.0%) had CES-D scores over 16, which are considered indicative of significantly depressed mood. FACIT fatigue scores were approximately normally distibuted with a mean and median of 31.

**Table 2:** Socio-Demographic Characteristics (N = 46)

**Characteristic**		**Mean ± Standard Deviation or N (%)**
**Gender**	Female	28 (60.9%)
**Age **(years)		72.3 ± 7.7
Education*	< highschool	11 (26.2%)
	highschool	21 (50.0%)
	> highschool	10 (23.8%)

*N = 42 for education

**Table 3:** Health Status Measures*

**Scale**	**Possible Scores**	**Mean ± Standard Deviation**	**Median**	**Range**
**WOMAC pain**	0–20	8.7 ± 3.9	8.0	0–18
**WOMAC physical function**	0–68	27.8 ± 11.6	25.0	7–48
**CES-D**	0–60	15.4 ± 8.9	14.5	2–44
**FACIT**	0–52	30.9 ± 9.0	30.5	14–47

* FACIT is scored so that lower scores indicate greater fatigue. All other scales are scored so that a higher score indicates greater symptom severity. WOMAC = Western Ontario and McMaster Universities Osteoarthritis Index; CES-D = Center for Epidemiologic Studies Depression Scale; FACIT = Functional Assessment of Chronic Illness Therapy Fatigue Scale

Five main themes were identified to summarize the focus groups: fatigue characteristics, factors affecting fatigue, impact of fatigue, methods of coping with fatigue and discussions about fatigue with others.

### Theme 1: Fatigue characteristics

Most participants indicated that they had experienced fatigue; they used a variety of terms to describe their fatigue, including "*tired*", "*exhausted*", "*worn out*", "*weakness*" and "*loss of energy*". One participant characterized fatigue as "*coming up against a brick wall*" and another said it was like "*somebody threw a pail of water over my head*". The majority of participants indicated that they felt fatigue and sleepiness were different. One stated, "*I think it's just a complete exhaustion and ... it's not like you're going to sleep but you just have to sit down and you just can't keep going*." Another said, "*Yeah, I felt the same way, when I get really fatigued it's not that I feel I'm tired, but I feel that if I don't go lie down, I'll fall down*." Several participants indicated that they felt that there were different types of fatigue. The terms 'mental' and 'physical' fatigue were used. The perceived differences between these were described by one participant: *"...physical fatigue is associated more with, I'd say, aches and pains in their limbs and muscles and backs and legs and so on...But the mental fatigue is something much different. You just feel absolutely drained out and you can't focus ...It does affect your thinking and how you approach different things and all of that, your ability to understand things. I think mental fatigue is much worse than physical fatigue. Physical fatigue you can get over it in a short time, but mental fatigue it may stay with you 2 or 3 days, maybe longer*." Several participants also distinguished what they felt was arthritis-related fatigue from their other experiences with fatigue, as illustrated by the following statement: "*I find that when you are fatigued from when you are doing work I mean you sleep, you wake up and you feel refreshed, but when you are fatigued from arthritis and you have rested or tried to rest, you're still fatigued when you wake up, you're still not there*."

### Theme 2: Factors affecting fatigue

Participants linked fatigue to a variety of factors. It was described as a consequence of aging and as due to various types of weather, including rainy, cold weather as well as heat, humidity and extreme weather changes. Participants disagreed with media reports and physicians who indicated that weather should not affect their arthritis symptoms. One participant questioned whether the relationship between fatigue and the weather might be related to depression, saying, "*I think sometimes too that when the weather's bad and the rain and you're really hurting and that tiredness comes on it's almost like part of a depression?*" Activity level, doing too much or too little, was also cited as a cause of fatigue. It was also linked to arthritis, in that it takes longer and requires more energy to do things because of arthritis and this then leads to fatigue. Pain was mentioned quite often as causing fatigue. One participant noted "..*I think it's wearing if you're in pain, it's very tiring*. " Additionally, medications taken to relieve pain were also said to cause fatigue: "*I do think that the pills we keep taking to keep the pain down...make you feel yucky and tired and draggy*."

Emotions and mood, including frustration, anger, stress and depression were additionally linked to fatigue by participants. However, mood was discussed in relation to arthritis generally, and specifically to pain, such that there seemed to be the suggestion of a pathway leading to fatigue, including the potential for feedback among symptoms. For example, one participant stated, "*I would say it's the pain comes first, then because of the pain you get tired. Pain is really debilitating for me anyway, and I get really tired and then because I'm so tired and because then I don't have the energy to do the things that I want to do, let alone the things that need to be done or should be done. Then I tend to get depressed or upset with myself and then I think it just begets more tiredness and it's a downhill slide*. " Similarly another participant said, "*I think the pain level ...grows and ... you feel fatigued, you feel stressed and stress brings on fatigue and ...then you get that stress tension*..." Some participants also stated that they experienced sleep difficulties at night due to arthritis-related pain and that this made them tired during the day.

### Theme 3: Impact of fatigue

Focus group participants indicated that fatigue had a substantial impact on their lives. We heard that fatigue was "*all consuming*" and "*totally overwhelming*". Emotional responses to fatigue were widely discussed. We heard that living with fatigue made participants irritable, cranky, frustrated, angry and depressed. For example, one participant explained, *" But the reverberation has [been] for me, more than anything else, for lack of a better word, depression. I'm not able...to focus, I'm not able to...work- I grew up on a farm. I've led a very physical life as well as a life of the mind too... But to me that fatigue thing, it is exhaustion like you said, you know, that...you can't put one foot in front of the other any more, but part of it is the exhaustion of the spirit or the exhaustion of the mind*." One participant compared pain and fatigue, stating, *"But do you find that the tiredness, like it's pain, it takes over our thinking and your thought process, and you almost shut down?" *Fatigue was also identified as affecting physical functioning. Participants indicated that it took them longer to do things, they required more rest and they were exhausted more easily. Several indicated that they had to limit or give up activites such as volunteer work, chores and lesiure or social activities. One female participant discussed at length the difficulty she had on a trip to the zoo with her husband, at a time when she felt her arthritis was particularly bad: "*I couldn't wait until I got to the next bench, I was having a terrible time, and he was quite exasperated. Well if you're going to be like that, why don't we just go home? Well no, that's not the point, the point is ... you're involving me in something that I can't partake in because I don't have the energy, I'm tired, I'm so fatigued I can't even think straight*." We also heard that for some participants, fatigue had affected their relationships. One participant stated, "*I think in relationships perhaps it's relationships of friends that maybe has suffered the most because by the time the evening comes around [I'm] too tired to go and do any visiting*."

### Theme 4: Methods of coping with fatigue

Participants discussed a variety of coping methods for dealing with fatigue, including resting, taking naps or hot baths, getting help with chores and using assistive devices. Pacing of activities was cited several times. For example, one participant noted, "*Yes, that's one thing, dividing jobs up instead of trying to do everything all at once, if you can do only one load of laundry today, fine." *Exercise was also mentioned as a means to reduce fatigue. One participant explained, ".. *you feel one hundred percent better really so good just to lay on the floor and just to do minor exercises just for fifteen, twenty minutes, and you'd be amazed at the energy you get after that." *Participants also discussed distracting oneself, visiting family and friends and cognitive ways of coping, including the acceptance of fatigue. One participant explained, "...*Because we find that if you're in cheerful company and there's something to laugh about, you can forget your fatigue and you can forget your pains*."

### Theme 5: Discussion about fatigue with others

When our focus group participants were asked whom they spoke to about their fatigue, there were relatively few responses; spouses were the only people mentioned. One participant stated, *"The only one I talk and compare notes [with] is with my husband. Then I say... you feel the same way, then I must be normal, you know you wonder... is it my imagination or is it normal, or is there something wrong with me ...I worry about when I don't know what's going on." *A male participant similarly stated that he only spoke to his wife and that, *"Nobody else wants to hear about it."*

## Discussion

Although generally not perceived as an important symptom in OA, the majority of our focus group participants with lower extremity OA clearly indicated that they had experienced notable fatigue. They made distinctions between what they perceived as arthritis-related fatigue and other experiences with fatigue, linking fatigue to OA pain and mental health and suggesting close inter-relationships among these symptoms. They indicated that fatigue had made a significant impact on their lives, affecting mental health and physical functioning and causing them to limit or give up activities.

Although the primary goal of our study was to collect qualitative data on fatigue in OA, we did collect quantitative data on the extent of fatigue experienced by participants in the previous week using the FACIT fatigue scale. The mean score of 30.9 (± 9.0) for our participants is approximately equal to mean scores of RA patients at baseline in two clinical trials (29.2 ± 11.1, 27.9 ± 11.0) [31]. It is also between baseline clinical trial scores of anemic (23.9 ± 12.6) and non-anemic (40.9 ± 9.8) cancer patients (higher scores = less fatigue) [11]. Although there are differences between these populations, these relative scores appear to support our qualitative findings that participants experienced a salient amount of fatigue.

Perhaps one of the reasons fatigue has largely been ignored in OA, is that OA is generally viewed as non-inflammatory and therefore unlikely to be associated with fatigue. However, this viewpoint negates the substantial body of literature that supports the links our focus group participants made between pain, mental health and fatigue. A 2003 structured, evidence-based review [36] concluded that there is an association between pain and fatigue and that the available evidence suggests that it may be an etiological relationship. Similarly, associations between mental health and fatigue have consistently been shown to be quite strong, especially for depression [20,37-39]. In OA specifically, Wolfe et al [20] found that pain was the strongest predictor of fatigue in OA, explaining 25% of the variance in fatigue scores in multivariate regression analyses. Depression was also a major predictor and was almost three times more common in persons with significant fatigue.

Several participants in our focus groups discussed sleep difficulties when asked about factors affecting fatigue, which is in accord with previous research [20]. Although most participants had indicated that they believed there was a difference between sleepiness and fatigue, some used the word "tired" when discussing sleep problems. This term is somewhat ambiguous in that it could refer to either sleepiness or fatigue. Pigeon et al [9] suggest defining sleepiness as drowsiness, sleep propensity, and decreased alertness, and fatigue as weariness, weakness, and depleted energy. The use of the word "tired" is a potential limitation of several available fatigue measures, including the FACIT.

Some of our focus group participants suggested that there were different types of fatigue, using the terms mental and physical fatigue. There is literature to suggest that there are multiple manifestations or dimensions of fatigue, including physical/neuromuscular, emotional/affective and mental/cognitive components [40], with varying predictors and consequences. To our knowledge, the potential for multiple dimensions of fatigue in OA has not yet been investigated.

A notable proportion, 37%, of our focus group participants had scores on the CES-D suggestive of significantly depressed mood, using the standard score cut-off of 16. There are challenges to measuring depression in the elderly [41] and in those with chronically painful conditions [42,43] such as the common inclusion of somatic symptoms in depression measures. Additionally, measures of depression include items that are included in measures of fatigue. Although fatigue and depression frequently occur together, fatigue is neither sensitive nor specific to the diagnosis of depression [38] and studies have demonstrated that fatigue and depression are distinct [44,45]. It is not possible to discern from our focus groups if depression or fatigue scores were inflated due to these issues. However, we do feel based on our participants' discussions that the fatigue they described was not solely a symptom of depression.

Co-morbidity is also unlikely to explain our study findings. Potential participants with co-morbidities closely linked to fatigue or pain were ineligible. Further, all but 5 participants had been part of an ongoing cohort study for the previous 7–9 years and by necessity of their continued participation are likely to be relatively healthy. This is supported by data collected in the year prior to the focus groups in which 54% (n = 22) of the 41 focus group participants with available data reported no other health conditions. Co-morbidities reported by the remaining participants were diabetes, hypertension, heart and lung problems and 27% of participants reported only one of these. Obesity is also a potential contributing factor to fatigue [46] and our cohort data indicate that of the 37 focus group participants with available height and weight data, 17 were obese. This is typical of the hip and knee OA population and we felt that any further exclusions would unduly jeopardize the generalizability of our findings.

A strength of our study is the use of participants recruited from a population-based cohort. The majority of research on fatigue in OA has utilized rheumatology clinic samples, which represent a small fraction of people with OA. However, as is the norm in qualitative research our findings are based on a small number of participants and should be replicated, although our participants do reflect the demographics of the hip and knee OA population. Additional qualitative and quantitative research will be needed to further clarify the role of fatigue in OA.

## Conclusion

Participants in our focus groups clearly described experiencing significant amounts of fatigue and indicated that it had a substantial impact on their lives. They highlighted emotional consequences and impacts on daily activities such as chores, as well as leisure and social activities. Better understanding the ways in which arthritis leads to disability and participation restriction has the potential to lead to new management strategies and to improve quality of life for those living with OA. Our findings represent an early step in increasing our understanding of fatigue in OA and support the small body of literature that suggests it may be an important symptom. Further research will be required in order to fully substantiate the role of fatigue and the associated clinical implications in OA as well as the relative contributions of factors discussed by our participants such as pain and depression, as well as obesity and co-morbidity. This will require research that incorporates larger sample sizes and longitudinal quantitative data. Many individuals living with arthritis never discuss their condition with their physicians and, although our participants discussed a variety of coping methods for fatigue, they did not generally discuss their fatigue with others. Individuals living with OA need to be encouraged to discuss all of their symptoms with their physicians, so that they are more likely to receive appropriate clinical attention.

## Competing interests

The authors declare that they have no competing interests.

## Authors' contributions

JDP analyzed and interpreted the study data and wrote the manuscript. GAH and EMB secured funding for the project, designed the study and contributed to the manuscript. MRF collected and analyzed study data and contributed to the manuscript. AJW collected study data and contributed to the manuscript. All authors read and approved the final manuscript.

## Pre-publication history

The pre-publication history for this paper can be accessed here:


